# Investigating topic modeling techniques through evaluation of topics discovered in short texts data across diverse domains

**DOI:** 10.1038/s41598-024-61738-4

**Published:** 2024-05-25

**Authors:** R. Muthusami, N. Mani Kandan, K. Saritha, B. Narenthiran, N. Nagaprasad, Krishnaraj Ramaswamy

**Affiliations:** 1 Department of Computer Applications, Dr. Mahalingam College of Engineering and Technology, Pollachi, Tamil Nadu India; 2 Department of Mechanical Engineering, P.A. College of Engineering and Technology, Pollachi, 642002 Tamil Nadu India; 3 Department of Mathematics, P. A. College of Engineering and Technology, Pollachi, Tamil Nadu India; 4 Department of Mechanical Engineering, Karpagam Academy of Higher Education, Eachanari, Coimbatore, 641021 Tamil Nadu India; 5 Department of Mechanical Engineering, ULTRA College of Engineering and Technology, Madurai, 625104 Tamil Nadu India; 6 Centre for Excellence-Indigenous Knowledge, Innovative Technology Transfer and Entrepreneurship, Dambi Dollo University, Dambi Dollo, Ethiopia; 7 Department of Mechanical Engineering, College of Engineering and Technology, Dambi Dollo University, Dambi Dollo, Ethiopia

**Keywords:** Probabilistic topic model, Non-probabilistic topic model, Short-texts, Topic discovery, Topic evaluation, Clustering methods, Engineering, Materials science, Mathematics and computing

## Abstract

The online channel has affected many facets of an individual's identity, commercial, social policy, and culture, among others. It implies that discovering the topics on which these brief writings are focused, as well as examining the qualities of these short texts is critical. Another key issue that has been identified is the evaluation of newly discovered topics in terms of topic quality, which includes topic separation and coherence. A topic modeling method has been shown to be an outstanding aid in the linguistic interpretation of quite tiny texts. Based on the underlying strategy, topic models are divided into two categories: probabilistic methods and non-probabilistic methods. In this research, short texts are analyzed using topic models, including latent Dirichlet allocation (LDA) for probabilistic topic modeling and non-negative matrix factorization (NMF) for non-probabilistic topic modeling. A novel approach for topic evaluation is used, such as clustering methods and silhouette analysis on both models, to investigate performance in terms of quality. The experiment results indicate that the proposed evaluation method outperforms on both LDA and NMF.

## Introduction

Media news items, statuses, weblog snapshots, Twitter postings, question/answer combos, and other short texts also seem to be vital resource. The primary features of these brief texts include a finite number of vocabulary within a text, as well as the adoption novel and colloquial vocabulary, definitions and utilizations of phrases that could vary dramatically relying on the publishing, and the minimal length of postings, such as a Twitter. Because of the prevalence of short text as in genuine life situations, short text research has gained traction in recent years^1–5^. To automatically analyze these massive numbers of messages and extract information, a powerful text processing technique is required. In the field of conventional computational linguistics, a topic modeling approach is highlighted as an important strategy enabling conceptual comprehension of text content^6,7^. Topic models can be classified into two groups based on their techniques: probabilistic approaches and non-probabilistic techniques. Latent semantic indexing (LSI)^8^ and non-negative matrix factorization (NMF)^9^ are two popular non-probabilistic techniques. The common probabilistic approaches are probabilistic latent semantic analysis (PLSA)^10^, latent Dirichlet allocation (LDA)^11^ and variational autoencoder (VAE)^12^. A handful of studies have looked into the use of short texts for topic modeling. In terms of research, topic models inevitably extract topics from the text, revealing a hidden relevant context in a sequence of textual data, and thus an identified challenging problem is evaluating the discovered topics in terms of quality.

Shi^13^ suggested a linguistic nonnegative matrix factorization approach that relies on NMF and incorporates phrase perspective semantic linkages to find themes for short texts. Xu^14^ created the LDA model, which recommends themes for a rigorous analysis of the Douban online review. Albalawi^15^ evaluated the efficacy of various topic modeling strategies using short textual datasets and standard numerical performance measures including recall, accuracy, F-score, and topic coherence. Qiang^16^ employed self-aggregation, Dirichlet multinomial mixture, and global word co-occurrences to examine topic modeling for short texts.

Fei^17^ developed a novel approach for multi-label emotion classification using a Latent Emotion Memory (LEM) network. The LEM network consists of two main components: a Latent Emotion Distribution (LED) module and a Classification module. The LED module is responsible for learning the latent emotion distribution of a given sentence without external knowledge. It does this by using a memory-augmented neural network that can capture the context information related to each emotion. The Classification module then uses the learned LED to predict the emotions present in the sentence.

Fei^18^ developed a novel method to multi-label emotion classification using a topic-enhanced capsule network. To enhance multi-label emotion classification performance, the model combines the advantages of topic modeling and capsule networks.

Wu^19^ built a complex system that involves several components, such as visual and textual scene graph generation, cross-modal graph construction, graph information bottleneck principle, and multimodal topic modeling with multimodal neural relation extraction dataset (MNRE).

Wang^20^ developed Topic-Guided Variational Auto-Encoder (TGVAE) generative model that learns to generate text by capturing the underlying topics in the data. It is an extension of the Variational Auto-Encoder (VAE) that incorporates a topic module to learn the topic distribution of the input text. Its ability to extract the underlying topics from the input data and turn it into coherent and meaningful text has been demonstrated. Many natural language processing tasks, including sentiment analysis, machine translation, and text summarization, can be accomplished with it.

Despite these efforts, evaluating the quality of acquired topics and how many should be instructive has become challenging. As a result, it's illogical to know the qualities of each model and how they can be useful for a certain application. Purity, adjusted rand index, perplexity, F-Measure, topic coherence, and normalised mutual information have all been used to evaluate topics and models. They don't take into account the quality of the topics covered by each model. The suggested evaluation strategy examines the quality of topics using clustering methods and silhouette analysis in topic models LDA and NMF^21^.

Despite the fact that it is a bag of word vector interpretation of a dataset, the NMF-based model learns topics by deliberately declining the word vector across dual low-dimensional feature vectors that have inherited to clustering feature from nature^22^. Since the LDA generative statistical model lacks clustering features by nature, hierarchical clustering techniques were used to build agglomerative clusters on a variety of themes^23–25^.

The following is a summary of the contributions to this research work.

- Created topic models for two short text datasets, SemEval 2016 and BBC News, employing latent Dirichlet allocation (LDA) as a probabilistic topic modeling method and non-negative matrix factorization (NMF) as a non-probabilistic topic modeling method. The developed topic models led to the discovery of new topics.
- A performance of LDA and NMF topic models on identified topics was investigated in terms of quality, employing clustering methods and silhouette analysis.
- According to the results obtained, the proposed evaluation approach outperforms on both the LDA and NMF topic models.

## Related work

The experimental work on topic modeling with clustering on short texts is a challenging study field with few recent studies.Yan et al.^22^ proposed the biterm subject model, an innovative approach for analyzing topics in lexical items. This model learns topics by analyzing the production of term co patterns through the entire collection. By quantitative evaluation, the clustering performances of this model and LDA have been compared with three standard metrics such as adjusted rand index, purity and the normalized mutual information. By qualitative evaluation, the quality of topics discovered by the two topic models was studied based on cosine similarity using a greedy algorithm. Quan et al.^26^ presented a self-aggregation oriented topic model (SATM) by extending the basic assumption of a standard topic model, i.e., LDA with the integration of clustering for short and sparse texts. Two metrics were used in the assessment: pointwise reciprocal information to calculate a topic's coherence and the purity criterion for clustering evaluation.

Shi et al.^27^ proposed an NMF-based model that incorporates the word context semantic correlations to discover topics for short texts, referred to as semantic-assisted nonnegative matrix factorization. In the quantitative evaluation of the model, the pointwise mutual information (PMI) score was calculated to measure the topic coherence. Blair et al.^28^ addressed the use of topic models aggregation to improve the coherence for social networking subjects. The topic coherence was calculated by computing word co-occurrences, mutual information, and normalised Google distance. Yi et al.^29^ proposed a topic model for concise messages focused on regularized NMF. Topic coherence is used to assess the discovery of topics from a corpus. Topic alignment is reliant on term co-occurrence again from the outside repository and is being found to correlate with individual assessments and has high inference performance. The normalized pointwise mutual information is used to compute the co-occurrence counts. Nasim et al.^30^ used Urdu tweets to do an experimental test of clustering techniques. Tweets are used to retrieve functions including sentence and word embedding, TF-IDF aspects, and clustering is performed using three main techniques: Affinity Propagation, K-Means, and Bisecting K-Means. Furthermore, the appraisal metrics Rand Index, Purity, and the adjusted mutual information were used in this work to evaluate cluster consistency.

All of the abovementioned works have been performed on topic modeling in which purity, NMI, ARI, and PMI have mostly been used as evaluation metrics. In this work, clustering methods and silhouette coefficients for the evaluation of topic modeling in terms of the quality of topics are proposed.

## Materials and methods

### Data preparation

Two benchmark datasets have been included in this research investigation: i. SemEval 2016 (Task 6), and ii. *BBC News.*

#### Dataset 1: SemEval 2016

An analysis was conducted using a benchmark dataset, SemEval 2016 (Task 6), which consisted of 4870 tagged English tweets regarding five major aspects, including atheism, climate change, feminist movement, Hillary Clinton, and legalization of abortion in the United States. Such tweets were about individuals, entities, policy initiatives, trends, and brands. Individuals often share their opinions on various target groups through comments on websites such as online forums, blogs, Twitter, YouTube, and Instagram. This analysis made use of the Task ‘A’ dataset (training set, 2914 tweets).

#### Dataset 2: BBC News

A public dataset acquired from the BBC News website, it contains 2225 documents pertaining to topics in five key themes from 2004 to 2005, including business, entertainment, politics, sport, and technology.

### Data preprocessing

Data preprocessing enables the construction of moderate classification tasks while also reducing computing overhead. On datasets, the relevant preprocessing procedures were used: eliminate the "RT" (retweet), urls, tags, punctuation, white spaces, stop words, and convert text to lower case. Furthermore, several repeated terms in the datasets were included in the list of stop words, such as "semst", "semest" in a dataset, SemEval 2016 and "say", "would" in a dataset, BBC News. The document-term matrix (DTM) was computed after preprocessing to build the topic model.

### Experimental setup of LDA

The introductory defines topic modeling is "eventually determining the topics in a set of texts." The recessed feature is the quantities of topics throughout text and phrases per theme. Inferring the underlying topic composition from the investigated texts is the most significant inference task in topic modeling. According to Blei^11^ and Muthusami^31^, the topic model such as LDA has been implemented in this research on both datasets, SemEval 2016 and BBC News with the parameters of the prior dispersals *α* = 10/k and *ϕ* = 0.1.

The assessment was then done on quantitative methodology of the fitted topic model, namely LDA, to derive the per-topic-per-term probability known as ("beta") on datasets. The model then determines the likelihood how this phrase could be created by this topic for each permutation. The assessed themes in a text and the assessed terms for a topic are represented below.

The top ten most relevant phrases for ten topics of fitted model LDA on dataset 1, SemEval 2016, are presented below.

**Table:** 

Topic 1 (atheism)	Topic 2 (feminist movement)	Topic 3 (legalization of abortion)	Topic 4 (hillary clinton)	Topic 5 (climate change is concern)
Change	God	Life	Know	Time
Today	Will	Abortion	Woman	People
Good	Equality	Nights	See	Get
Love	Going	Human	Scotus	Need
Day	Always	Much	Say	Like
Nothing	Need	Pretty	President	Never
Work	Feminist	Like	Marriage	First
World	Look	Prolifeyouth	Best	Better
Jesus	Every	Bad	Well	Stop
Believe	Must	Care	May	Male

**Table:** 

Topic 6 (feminist movement)	Topic 7 (legalization of abortion)	Topic 8 (legalization of abortion)	Topic 9 (hillary clinton)	Topic 10 (hillary clinton)
Women	Can	Just	Don't	Hillary
Men	People	One	Want	New
Feminism	Even	Right	Feminists	Doesnt
Way	Like	Now	Love	Really
Feminist	World	Man	Gamergate	Think
Live	Wants	Vote	Made	Truth
Make	Take	Let	Give	Gender
Without	Wakeupamerica	Children	Tcot	Clinton
Country	Pro	Make	Still	Peace
Unborn	Babies	Many	Hillaryclinton	Win

The ten topics from the dataset, SemEval 2016, may be interpreted from the findings. For example, the most prevalent terms in topic 1 are "love," "effort," "Jesus," and "believe," all of which imply atheism. The most commonly used phrases in topic 2 are "equality," "need," "look," and "feminist," implying that this topic reflects a feminist viewpoint. Topic 3's most prevalent keywords include "abortion," "human," and "rights," implying that this topic represents the legalisation of abortion perspective. The most prevalent phrases in topic 6 are "women," "without," "way," "men," and "living," implying that this topic expresses a feminist perspective.

The top ten most relevant terms for ten topics of fitted model LDA on dataset 2, BBC News, are shown below.

**Table:** 

Topic 1 (politics)	Topic 2 (technology)	Topic 3 (entertainment)	Topic 4 (entertainment)	Topic 5 (business)
Labour	People	People	Film	Year
Election	Many	Can	Best	Last
Blair	Can	Mobile	Show	Sales
Government	Net	Music	Also	Growth
Party	Software	Also	Director	Economy
People	Users	New	Won	Economic
Minister	Internet	Digital	Year	Market
Brown	Online	Games	One	Rise
Tax	Information	Phone	Star	Fig.s
Public	Use	Technology	Top	Interest

**Table:** 

Topic 6 (business)	Topic 7 (sport)	Topic 8 (sport)	Topic 9 (politics)	Topic 10 (politics)
Company	England	World	Government	Former
Deal	Game	Year	Home	Two
Firm	Half	Two	New	Last
New	Six	First	Law	Also
Group	First	Last	Say	Action
Financial	Back	Game	Bill	Decision
Money	Side	Set	Police	Three
Also	Time	Second	Secretary	Court
Shares	Ireland	Time	Plans	One
Companies	Win	Open	Also	Later

The findings indicate the 10 topics of the fitted model LDA from the dataset, according to BBC News. For example, topic 1's most common terms are "labour," "government," "election," "blair," "party," "minister," and "people," showing that this topic is about politics. The most common terms in topic 3 are "people," "mobile," "music," "digital," "top," and "games," implying that this topic is about entertainment. The most common terms in topic 5 are "year," "growth," "economy," "economic," "market," and "increase," all of which pertain to business. The most common terms in topic 7 are "game," "first," "england," "win," and "team," showing that this topic is about sports.

### Experimental setup of NMF

The standard NMF problem is expressed in terms:

Make X denote the n x p nonnegative matrix, (i.e., xij > 0, X ≥ 0), and the factorization rank, r ≥ 0, is an integer. The NMF entails identifying an estimate as given in Eq. (1).

1 $${\text{X }} \approx {\text{ WH}},$$

In which W and H are nonnegative arrays of size n x r and r x p, accordingly. In reality, the permutation level r is always characterized by the fact that r <  < min (n,p). The aim of such an option is to quantify and partition the details in X into r variables: the indexes of W. These variables are referred to differently relying on the implementation field: base objects, metagenes, and target sensors. In this article, the terms base vector or metagenes are used to adhere to array W, and blend coefficient matrix and metagene expression profiles are used to refer to array H.

As seen in Eq. (2), the key approach to NMF estimates arrays W and H, as a given problem.

2 $$\mathop {\min }\limits_{W,H \ge 0} \frac{{\left[ {{\text{D}}\left( {{\text{X}},{\text{ W H}}} \right){ } + {\text{ R}}\left( {{\text{W}},{\text{ H}}} \right)} \right]}}{{ = F\left( {W, H} \right)}}$$

In which D is a depletion process that describes the approximation's accuracy. As seen in Eq. (3), typical depletion process is dependent on the Euclidean or the Frobenius distance.

3 $$D:A, B \to \frac{Tr(A{B}^{t})}{2}=\frac{1}{2} \sum_{ij}{\left({a}_{ij}- {b}_{ij}\right)}^{2}$$

or the Kullback–Leibler divergence as given in Eq. (4),

4 $$D:A, B \to K L (A||B)=\sum_{i,j}\left({a}_{ij}{\text{log}}\frac{{a}_{ij}}{{b}_{ij}}- {a}_{ij}+{b}_{ij}\right)$$

R is an alternative convolution method that is described to impose desired traits on matrices W and H, such as crispness or sparseness. In particular, NMF methodologies find a solution (2) adaptively by constructing a series of arrays (Wk, Hk) that affect the value of the optimal solution F on every stage. They vary in the optimization methods used to calculate the changes for F, which response to certain discrepancies in the definition of (Wk, Hk).

In this work, the NMF algorithm, Lee and Seung method^32^, in which simple multiplicative updates are used based on Euclidean distance, as given in Eqs. (5) and (6).

5 $${H}_{kj} \leftarrow {H}_{kj} \frac{({W}^{T}V{)}_{kj}}{({W}^{T} W H{)}_{kj}}$$

6 $${W}_{ik} \leftarrow {W}_{kj} \frac{(V {H}^{T}{)}_{ik}}{({W H H}^{T}{)}_{ik}}$$

To begin the recursive step NMF methods were seeded (i.e., from a value of W_0_ and/or H_0_). Since here is not general quantification method and the task has a wide dimensions the option of iteration is crucial for generating useful performance. A most general propagation approach has been used as an arbitrary reference point, at which instances of W and/or H were taken from a homogenous dispersion normally in that same scope as the desired matrix's entrants, in which several iterations were conducted, also with a specific reference level which greatly improved the computational duration and obtained the required convolution. The top ten most relevant phrases for ten topics of fitted model NMF on dataset 1, SemEval 2016, are presented below.

**Table:** 

Topic 1 (atheism)	Topic 2 (feminist movement)	Topic 3 (hillary clinton)	Topic 4 (atheism)	Topic 5 (hillary clinton)
Dont	Right	Hillari	God	Hillaryclinton
Know	Equal	Clinton	Love	Want
Want	Now	Need	Bless	Cant
Like	Human	Get	Day	Even
Think	Unborn	Like	Pray	Billclinton
Best	Live	Campaign	Thank	Potus
Love	Life	Email	Believ	Lol
What	Scotus	Vote	Always	Manag
Need	Time	Support	Now	Wakeupamerica
Matter	Law	Tcot	May	Stophillari

**Table:** 

Topic 6 (feminist movement)	Topic 7 (legalization of abortion)	Topic 8 (legalization of abortion)	Topic 9 (atheism)	Topic 10 (atheism)
Equal	Can	Abort	God	Will
Gamerg	People	One	Always	Come
Hate	Prochoice	Life	Love	Time
Dear	Like	Just	Pray	Never
Get	Say	Make	Bless	Say
Men	Change	Get	Will	Always
Man	Take	Babi	Need	Get
Femin	Kid	Kill	Want	Day
Want	Cant	Human	May	Ever
Feminist	Think	Woman	Thank	Life

The findings can be used to analyse the 10 themes from the dataset SemEval 2016. For example, in topic 2, the most common phrases are "right," "equal," "human," and "scotus," all of which indicate feminist. Topic 4's most often used terms are "god," "love," "bless," and "pray," suggesting that this topic represents an atheist attitude. The most common terms in topic 8 are "abort," "babi," and "kill," showing that this topic supports the legalisation of abortion viewpoint.

The top ten most relevant phrases for ten topics of fitted model NMF on dataset 2, BBC News, are presented below.

**Table:** 

Topic 1 (politics)	Topic 2 (sport)	Topic 3 (politics)	Topic 4 (politics)	Topic 5 (business)
Labour	Game	Government	Court	Just
People	First	European	Told	Get
Election	England	New	Law	One
Blair	Win	Also	Two	Can
Government	World	Country	Action	Like
Party	Two	World	Police	Think
Minister	Back	Countries	Legal	Going
Public	Team	Foreign	Bill	Good
Brown	Half	Legal	Rights	Make
Law	Six	Can	Also	Best

**Table:** 

Topic 6 (entertainment)	Topic 7 (entertainment)	Topic 8 (technology)	Topic 9 (business)	Topic 10 (business)
One	Best	Can	Growth	Company
Number	Film	People	Economy	Firm
Best	Year	Also	Sales	Deal
Top	Doctor	Mobile	Last	New
Music	Also	World	Expected	Financial
First	Won	Technology	Economic	Chief
New	British	Net	Market	Shares
Show	Awards	Digital	Rise	Executive
World	Star	Use	Year	Offer
Year	Filims	New	Shares	Sales

According to dataset 2, BBC News, the findings highlight the ten topics of the fitted model NMF. Topic 1's most prevalent phrases, for example, are "labour," "people," "election," "blair," "party," "minister," and "public," indicating that this topic is about politics. The most often used phrases in topic 3 are "government," "european," "country," "world," and "legal," emphasizing that this is a political topic. Topic 7's most popular phrases are "best," "film," "year," "won," "british," and "awards," all of which are related to entertainment. Topic 10's most used phrases are "company," "firm," " financial," "chief," "shares," and "sales," indicating that it is about business.

## Evaluation

In this section, as discussed in Sect. 1 and Sect. 2, agglomerative hierarchical clustering algorithms were implemented on documents containing topics that were generated by the generative probabilistic topic model, i.e., LDA by which the results of topic modeling evaluate the topics in terms of quality, i.e., goodness in topics separation and topic cohesiveness.

**Figure 1:**
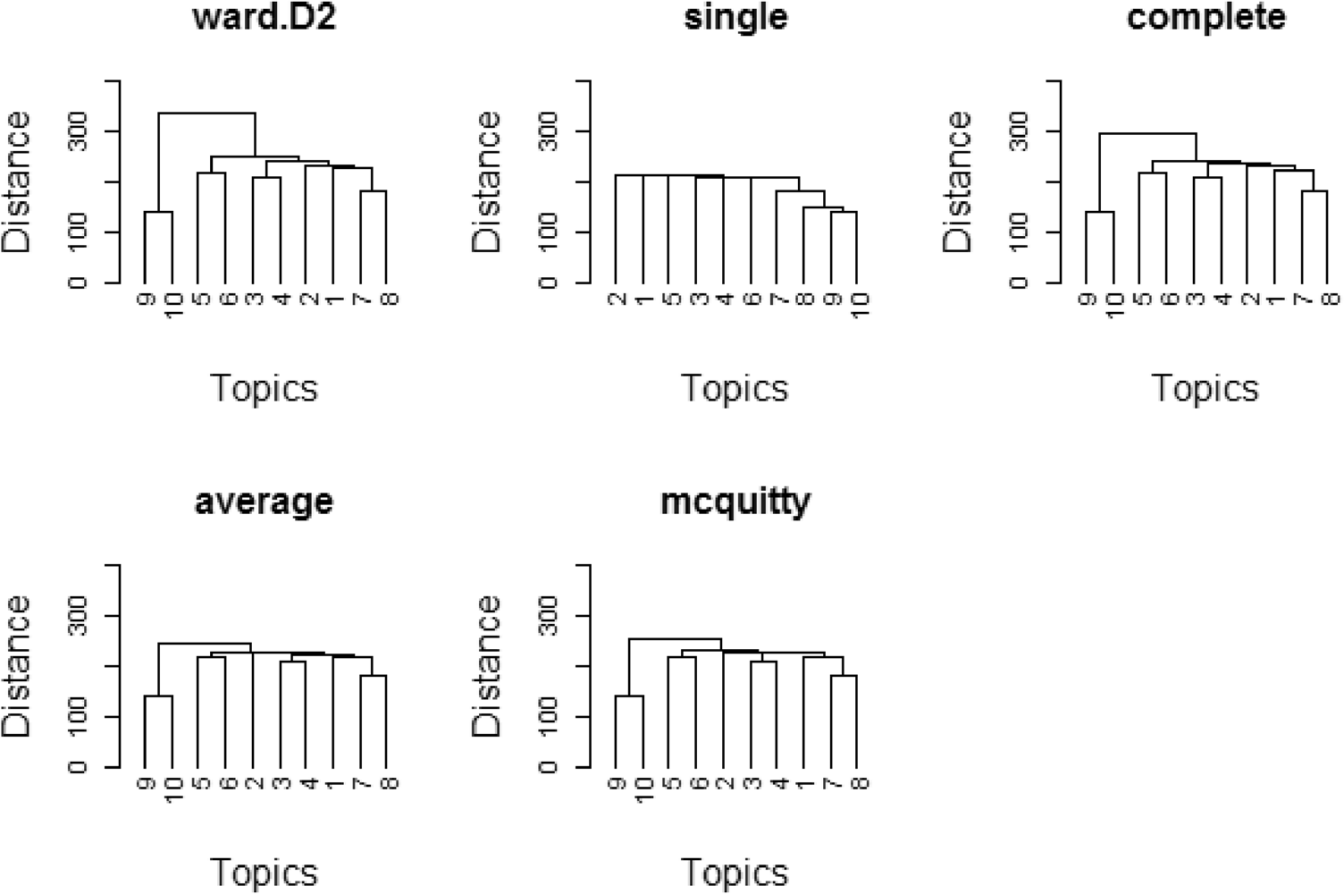
Dendrograms of various cluster linkage methods on fitted model LDA for 10 topics on dataset 1, SemEval 2016.

In this research uses hierarchical cluster approaches to test suited LDA topic model. A dendrogram is a hierarchical tree of groups generated by hierarchical methods. Hierarchical clustering methods were applied on generative topic models in the experimental work, such as LDA, which uses dissimilarities (similarities) or distances (Euclidean distance, city-piece (Manhattan) distance, gamma distance, cophenetic distance, and angle distance) among items when framing the clusters^33^. Similarities are indeed a collection of criteria which are drawn in there as conditions for classifying or confining items. These distances (similarities) are being located on a specific factor or several aspects, from each aspect stating a principle or requirement for classifying items. In this research, numerous cluster analysis linkage approaches were used, including single linkage (nearest neighbor), complete linkage (farthest neighbor), unweighted pair-group average (UPGMA), Ward's method, and the McQuitty method. The dendrogram of the LDA Gibbs sampling fitted model with four cluster methods is shown in Fig. 1: (i) Ward. D2 (ii) single (iii) complete (iv) Average (v). McQuitty.

**Figure 2:**
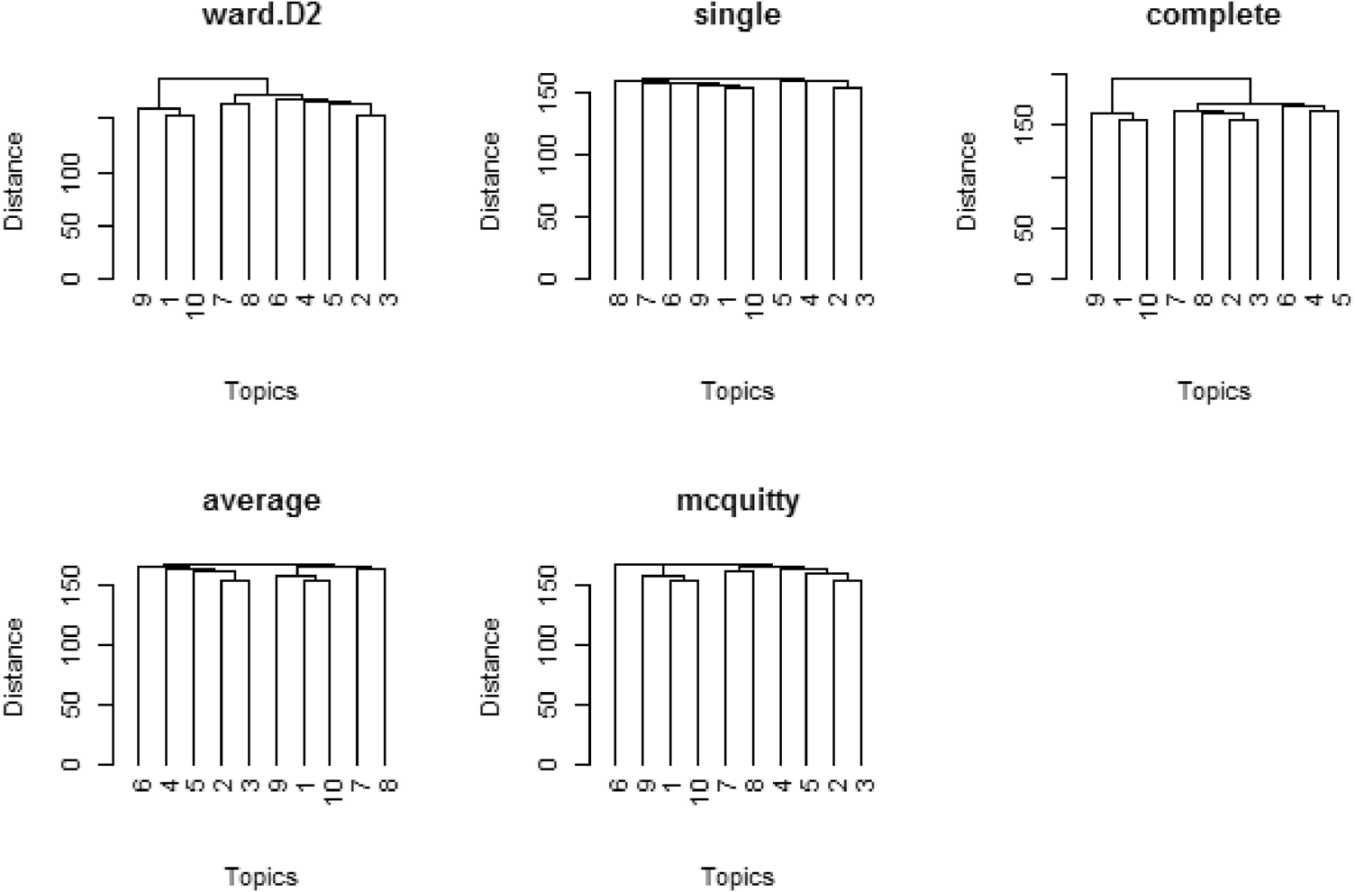
Dendrograms of various cluster linkage methods on fitted model LDA for 10 topics on dataset 2, BBC News.

Figure 2 portrays the dendrogram of the LDA-fitted model on dataset 2 with five groups of strategies, (i) Ward. D2 (ii). single (iii) complete (iv) average (v). McQuitty.

Figures 1 and 2 illustrate the relationship between the dendrogram and dissimilarity as evaluated among objects. Essentially, the level at which branches merge is related to their similarity. The five dendrograms are assessed by computing the agreement value using various distance and linkage methods in the LDA Gibbs sampling fitted model on dataset 1 and 2, in which the ‘McQuitty-link’ cluster method outperformed the angle distance method, i.e., the ‘McQuitty-link’ method. The agreement value is very close to 100%, which shows that the goodness of clustering and the topics discovered by this model have more cohesiveness.

**Figure 3:**
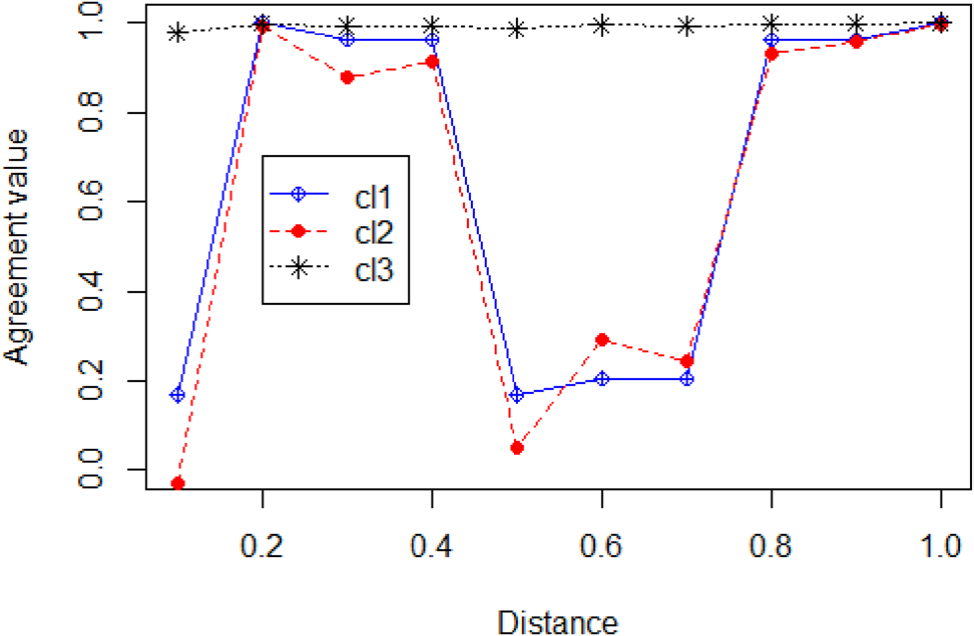
Shows the agreement value of the McQuitty linkage cluster method applying.

**Figure 4:**
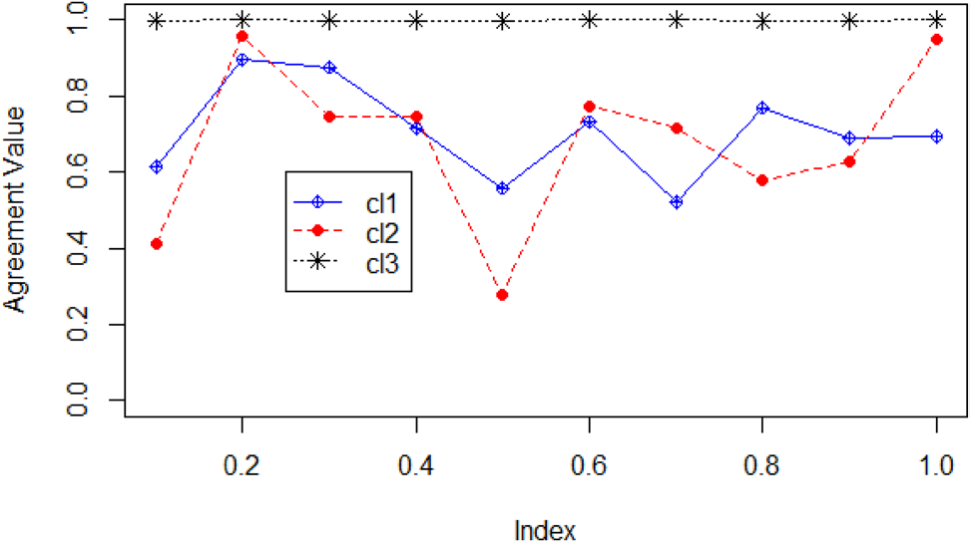
Shows the agreement value of the McQuitty linkage cluster method applying.

Figures 3 and 4 demonstrate the ‘McQuitty’ based clustering strategy using the gamma, cophenetic and angle distance methods on dataset1 and dataset 2, respectively.

various distance methods in the LDA topic model for 10 topics on dataset 1, SemEval 2016.

(cl1: gamma distance, cl2: cophenetic distance, cl3:angle distance).

Figure 3 illustrates that objects 9 and 10 have higher agreement values in the case of all three distance methods. The objects 2, 3, 4, 5, 6 and 7 have almost equal agreement values. These objects indicate the similarity among them. Objects 9 and 10 have a higher agreement value, which shows that the objects have higher intersimilarity, i.e., cohesiveness, which could be compared with the dendrogram based on the McQuitty linkage method (Fig. 1). There the level at which branches merge relates to their similarity. In the example, objects (i.e., topics) 7 and 8 are more similar to each other. In another case, objects 3 and 4 are more similar to each other. The same thing could be interpreted with human assessment of the retrieved topics by the fitted model LDA on dataset 1, SemEval 2016.

various distance methods in the LDA topic model for 10 topics on dataset 2, BBC News.

(cl1: gamma distance, cl2: cophenetic distance, cl3:angle distance).

Figure 4 illustrates that objects 1, 9 and 10 have higher agreement values in the case of all three distance methods. Objects 2, 3, 4, 5, 6, 7 and 8 have almost equal agreement values. These objects indicate the similarity among them, which can be compared with the dendrogram created based on the McQuitty linkage method (Fig. 2). In the example, objects (i.e., topics) 9 and 10 are more similar to each other compared to object 1. In addition, objects 3 and 4 are more similar to each other. The same thing could be interpreted with human assessment of the retrieved topics by the fitted model LDA on dataset 2, BBC News.

In an overview, the proposed technique was contrasted with another method, perplexity. Perplexity has been used as the most common method for assessing topic models^34,35^. In the case of the LDA, it is not so good because it is hard to comprehend, LDA works well when the topics are soft clustered. Sometimes it shows better on the topic model when learning works smoothly, but it shows very poor overall model quality. Therefore, the suggested method was better than other techniques, i.e., perplexity.

An experiment was further conducted on some hierarchical clustering validation methods for investigating the performance of the LDA model in terms of goodness of clustering, topic or cluster cohesion, isolated or separation and compactness of clusters for the optimal number of clusters identified using Dindex and Hubert statistic indices methods based on the silhouette width index method that was deployed. The dendrogram of the McQuitty linkage method was considered in further evaluation since it is comparatively the better method in LDA on both datasets, which has been proven in the experiment. The Dindex and Hubert statistic indices were implemented to identify the optimal number of clusters.

**Figure 5:**
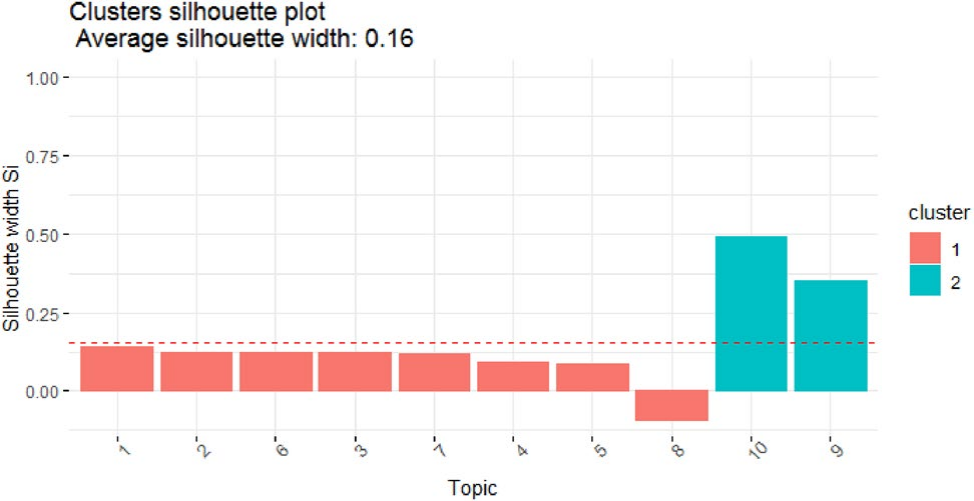
Clusters silhouette of fitted model LDA on dataset 1, SemEval 2016.

**Figure 6:**
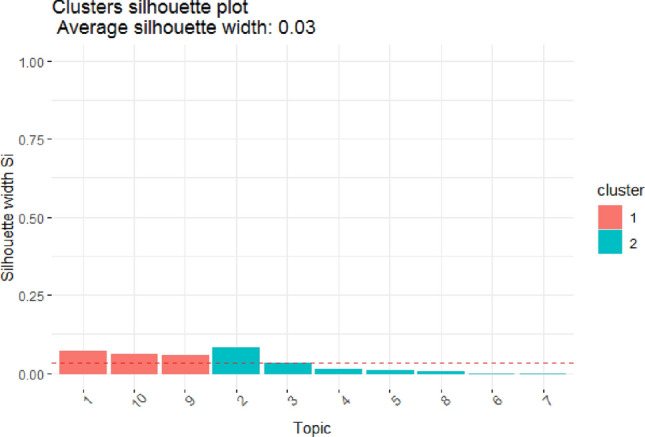
Clusters silhouette of fitted model LDA on dataset 2, BBC News.

The experimental results revealed that the optimum number of clusters for the LDA-fitted model was two on both datasets. The cluster dendrogram of the McQuitty method was addressed next on both datasets of the fitted model LDA by the implementation of the silhouette width index method with an optimal number of clusters as 2. The silhouette characterization signifies even if an inference was clustered and computes the relative interval between clusters. The silhouette Fig. represents how connected each point in one cluster is to points in neighboring clusters. The experimental results are shown in Figs. 5 and 6 and Table 1.

**Table 1:** Silhouette width of clusters in fitted model LDA on various datasets.

Dataset 1, SemEval 2016			Dataset 2, BBC News		
Topics	Cluster	Avg. sil_width	Topics	Cluster	Avg. sil_width
1,2,6,3,7,4,5,8	1	0.08956622	1,9,10	1	0.06305456
10,9	2	0.42051240	2,3,4,5,6,7,8	2	0.02009829
Mean Silhoutte width: 0.1557555			Mean Silhoutte width: 0.03298517		

From Fig. 5 and Table 1, it can be observed that on dataset 1, cluster 1 has a lower average silhouette width, i.e., 0.09 (9%), and cluster 2 has an average silhouette width, i.e., 0.42 (42%). There is a difference between both values, which indicates that the model is good in cluster separation. The inter-cluster objects relation is seen, i.e., the distance between the objects within the cluster. For example, if it takes cluster 2 (which has 2 objects or topics such as 9 and 10), the distance between specific 2 objects i.e., topics 9 and 10 is very small. Similarly, the distance between inter-objects of cluster 1 is very close, which indicates that the model is good in topic or cluster cohesion or compactness.

On dataset 2, according to Fig. 6 and Table 1, cluster 1 and cluster 2 have significantly lower typical silhouette distances, i.e., 0.06 (6%) and 0.02 (2%), respectively, and there is a discrepancy in both values, indicating that the model is good at cluster separation. If the inter-cluster objects relation is observed, i.e., the distance between the objects within the cluster, for example, if it takes cluster 2 (which has seven objects or topics such as 2,3,4,5,6,7, and 8), the distance between a specific two objects, i.e., topics 4 and 5, is the smallest; similarly, the distance between inter-objects of cluster 1 is very small, indicating that the model is good in topic or cluster cohesion or compactness.

The results reveal the topic evaluation of the agglomerative hierarchical clustering algorithms in the LDA topic model on both datasets outperforms in topics separation (maximizing inter-cluster distance) and topic or cluster cohesion (minimizing intra-cluster distance), i.e., good in terms of quality.

Furthermore, to evaluate the efficiency of the NMF model, the ideal number of clusters was determined and estimated with the clusters silhouette width of the fitted model NMF on various datasets.

**Figure 7:**
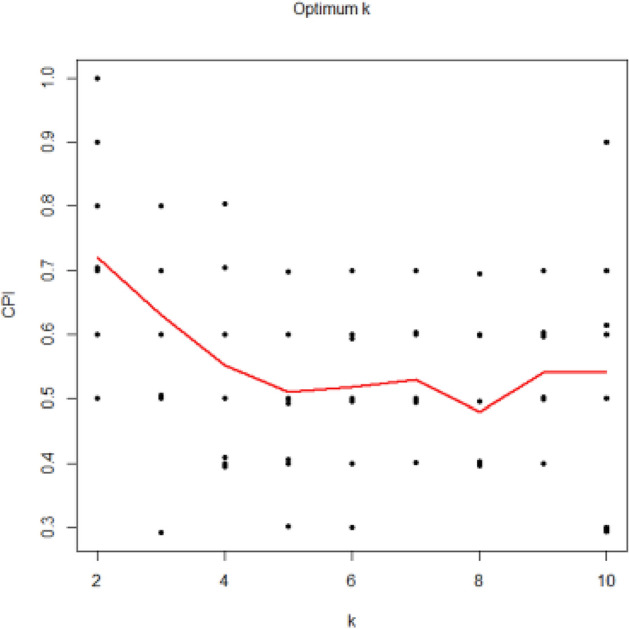
Shows the optimal number of clusters for the fitted model NMF on dataset 1, SemEval 2016.

**Figure 8:**
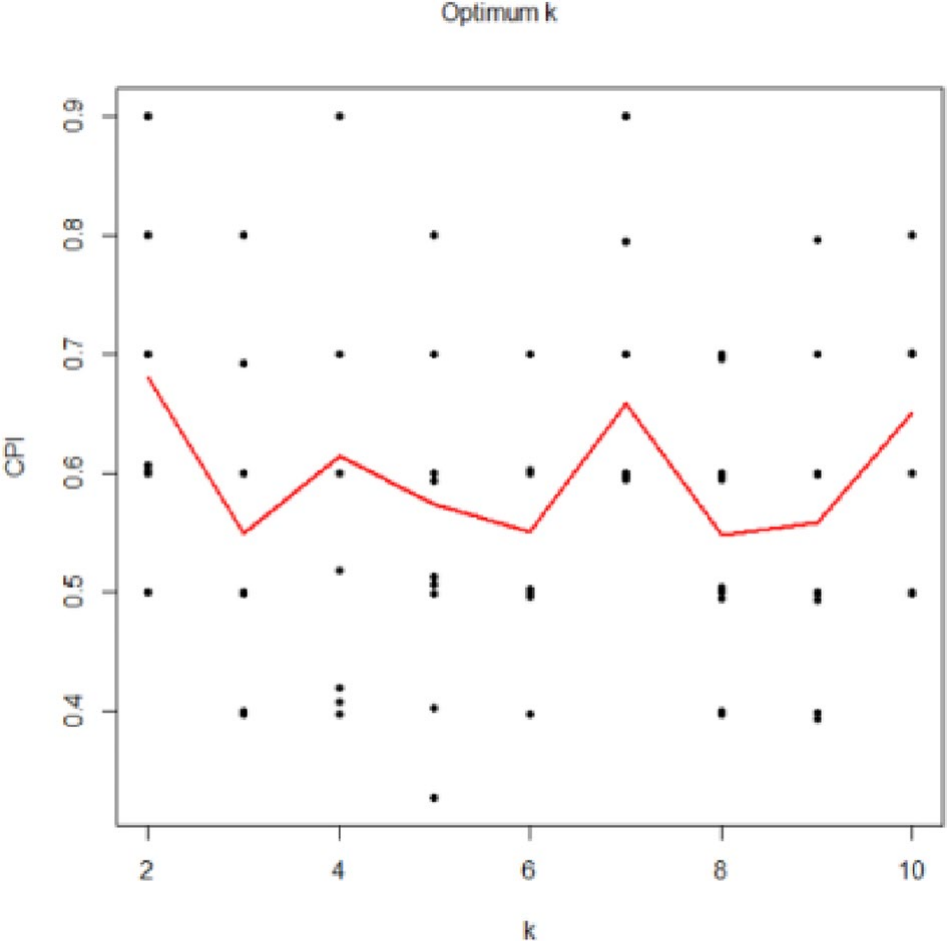
Shows the optimal number of clusters for the fitted model NMF on dataset 2, BBC News.

In the case of determining the optimal number of clusters in NMF, the intNMF algorithm was appropriately implemented on the test data to determine the clustering distributions of the samples in the test data, and the value of ‘k’ that consists in the optimum amount of the cluster prediction index (CPI) is assumed to be the appropriate clusters again for data. From Figs.7 and 8, the optimum number of clusters for the fitted model NMF on dataset 1, SemEval 2016 and dataset 2, BBC News are 2.

**Figure 9:**
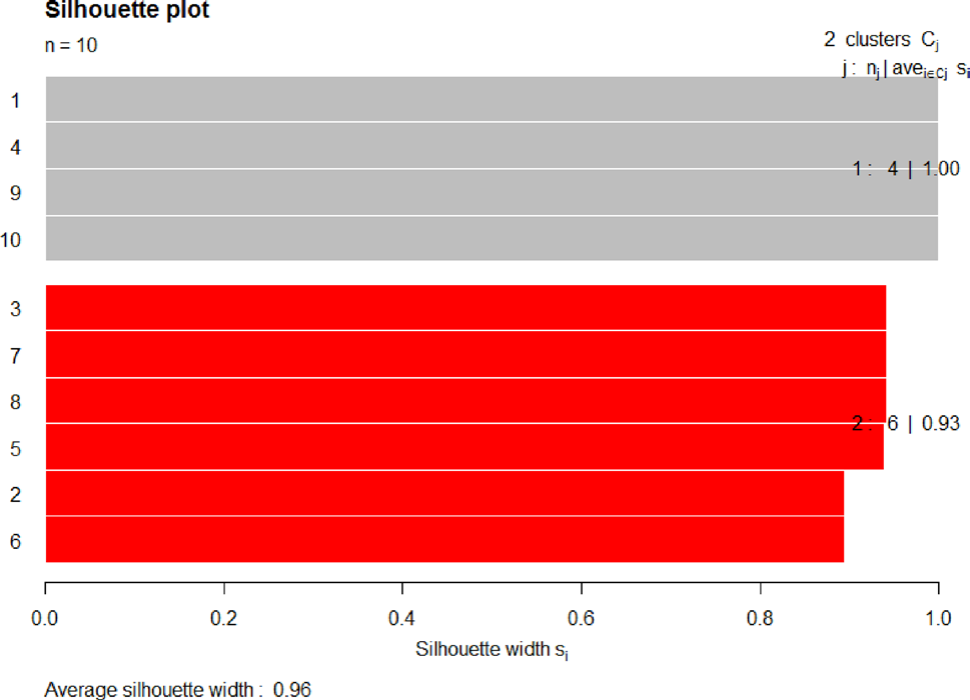
Display clusters silhouette of fitted model NMF on dataset 1, SemEval 2016.

**Figure 10:**
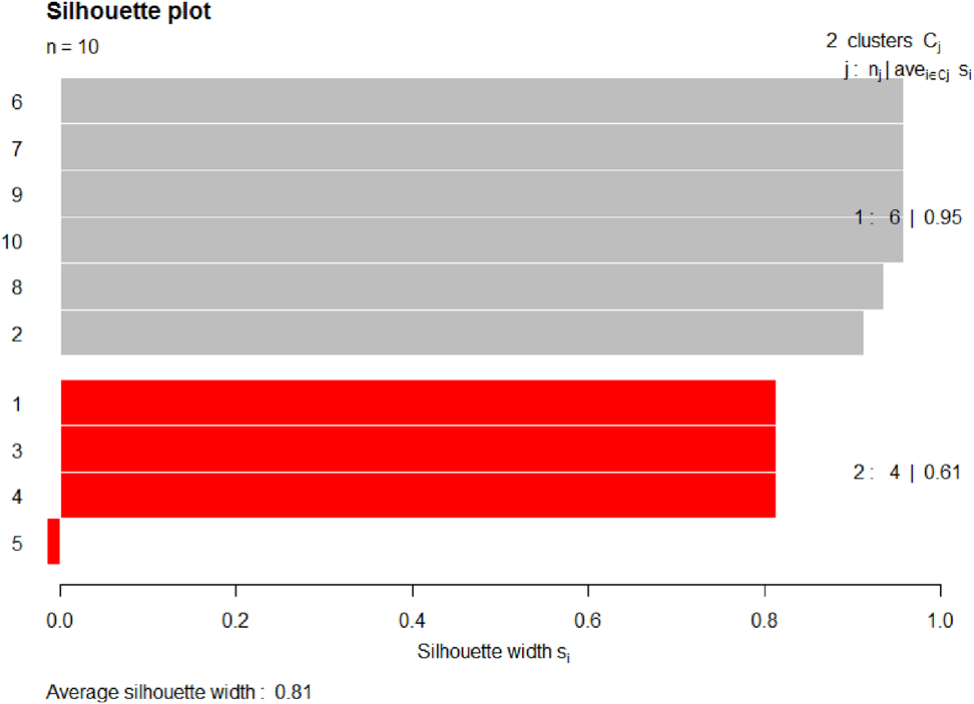
Display clusters silhouette of fitted model NMF on dataset 2, BBC News.

Figures 9 and 10 illustrate the clusters silhouette of fitted model NMF on dataset 1, #SemEval 2016 and dataset 2, BBC News, respectively. The details of the clusters, topics, average silhouette width and mean silhouette width of both datasets are given in Table 2.

**Table 2:** Silhouette width of clusters in the fitted model NMF on various datasets.

Dataset 1, SemEval 2016			Dataset 2, BBC News		
Topics	cluster	Avg. sil_width	Topics	cluster	Avg. sil_width
1,4,9,10	1	1.0000000	6,7,9,10,8,2	1	0.95
2,3,7,8,5,6	2	0.9257678	1,3,4,5	2	0.6056985
Mean Silhoutte width: 0.9554607			Mean Silhoutte width: 0.8101837		

From the results shown in Fig. 9 and Table 2, it can be observed that on dataset 1, clusters 1 and 2 both have maximum average silhouette widths, i.e., 1.00 (100%) and 0.92 (92%). There is a tiniest difference between both values, which indicates that the model is good in cluster separation. If the inter-cluster objects relation is seen, i.e., the distance between the objects within the cluster, for an example if it takes cluster 1 (which has 4 objects or topics, such as 1,4, 9 and 10), the distance between any two of the objects is the same. Similarly, the distance between inter-objects of cluster 2 is very close, which indicates that the model is good in topic or cluster cohesion/compactness.

From Fig. 10 and Table 2, on dataset 2, clusters 1 and 2 both have high average silhouette widths, i.e., 0.95 (95%) and 0.61 (61%). There is a normal difference between both values, which indicates that the model is good in cluster separation. If the inter-cluster objects relation is seen, i.e., distance between the objects within-cluster, for an example if it takes cluster 2 (which has 4 objects or topics such as 1, 3, 4 and 5) the distance between specific 2 objects i.e., topics 1 and 3, 3 and 4 are zero, and object 5 has a negative silhouette width. Similarly, the distance between inter-objects of cluster 1 is very low, which indicates that the model is good in topic or cluster cohesion/compactness.

The results reveal that the topic evaluation of the clustering methods in the NMF topic model on both datasets outperforms in topics separation (maximizing inter-cluster distance) and topic or cluster cohesion (minimizing intra-cluster distance), i.e., good in terms of quality.

The limits of this work include topic stability, the quantity of datasets used, and the types of models evaluated, all of which will be addressed in future work.

## Conclusion

In this research, an attempt is made to analyse short texts employing the topic model, latent Dirichlet allocation (LDA), probabilistic topic modeling, to find topics related to the concerned content. Since the LDA generative probabilistic model lacks clustering properties by convention, agglomerative hierarchical clustering methods were deployed on intent to generate various topics in the form of trees known as 'dendrograms.' Following that, an analysis was performed on those lists of dendrograms for evaluating the topic model, which possibly led to an investigation of the performance of the model by computing clustering validation method, silhouette coefficient.The proposed evaluation approach has been used in conjunction with the NMF, non-probabilistic topic model. The detailed research revealed that the LDA and NMF topic models performed better in terms of quality in the evaluation of topics using clustering methods on short texts.

In the future, short text data sets, such as those from the biological and applied sciences, will be taken into consideration by the proposed approach further in order to assess a more intricate framework for recommendation tasks that combines Neural Topic Models (NTM) with different structures including Variational AutoEncoder (VAE), embeddings and metadata.

## Data Availability

The datasets used and analyzed during the current study are available from the corresponding author on request.
